# Synthesis and Bio-Activity Evaluation of Scutellarein as a Potent Agent for the Therapy of Ischemic Cerebrovascular Disease

**DOI:** 10.3390/ijms12118208

**Published:** 2011-11-18

**Authors:** Li-Hua Qian, Nian-Guang Li, Yu-Ping Tang, Li Zhang, Hao Tang, Zhen-Jiang Wang, Li Liu, Shu-Lin Song, Jian-Ming Guo, An-Wei Ding

**Affiliations:** 1 Jiangsu Key Laboratory for High Technology Research of TCM Formulae, Nanjing University of Chinese Medicine, Nanjing, Jiangsu 210046, China; E-Mails: nanzhongyilihua@139.com (L.-H.Q.); linianguang@163.com (N.-G.L.); 15850534175@163.com (H.T.); tianruoxi@126.com (Z.-J.W.); liuli-44@163.com (L.L.); sshl2009@126.com (S.-L.S.); njuguo@njutcm.edu.cn (J.-M.G.); ltcmf@njutcm.edu.cn (A.-W.D.); 2 Department of Medicinal Chemistry, Nanjing University of Chinese Medicine, Nanjing, Jiangsu 210046, China

**Keywords:** scutellarein, scutellarin, synthesis, antioxidant activity, ischemic cerebrovascular disease

## Abstract

Scutellarein, the main metabolite of scutellarin *in vivo*, has relatively better solubility, bioavailability and bio-activity than scutellarin. However, it is very difficult to obtain scutellarein in nature compared with scutellarin. Therefore, the present study focused on establishing an efficient route for the synthesis of scutellarein by hydrolyzing scutellarin. The *in vitro* antioxidant activities of scutellarein were evaluated by measuring its scavenging capacities toward DPPH, ABTS^+•^, ^•^OH free radicals and its protective effect on H_2_O_2_-induced cytotoxicity in PC12 cells using MTT assay method. The results showed that essential point to the synthesis was the implementation of H_2_SO_4_ in 90% ethanol in N_2_ atmosphere; scutellarein had stronger antioxidant activity than scutellarin. The results have laid the foundation for further research and the development of scutellarein as a promising candidate for ischemic cerebrovascular disease.

## 1. Introduction

Cerebrovascular disease is a common and frequently-occurring disease that seriously endangers human health. It is one of the leading causes of death and disability worldwide, especially ischemic cerebrovascular disease, which is the most frequently prevalent [1]. Increasing evidence suggests a critical role of oxidative stress in ischemic cerebrovascular disease [2,3], and natural flavonoid antioxidants are well known as free radical scavengers [4]. Scutellarin (4′,5,6-trihydroxyflavone-7-glucuronide), the major anti-oxidant constituent in breviscapine extracted from Chinese herb of *Erigeron breviscapus* (vant.) Hand.Mazz., showed the effectiveness on dilating blood vessels, improving microcirculation, increasing cerebral blood flow, and inhibiting platelet aggregation since the 1970s [57]. In addition, it has been clinically used to treat acute cerebral infarction and paralysis induced by cerebrovascular diseases such as hypertension, cerebral thrombosis, cerebral haemorrhage in China since 1984 [8].

Although scutellarin has been clinically used for a long time, scutellarin has low water-solubility (just 0.16 mg/mL [9]) and lipid-solubility (log P = −2.56 in PBS at pH 4.2 [10]). Moreover, the bioavailability of scutellarin was very low; the absolute bioavailability in Beagle dog administered orally was rarely 0.4% [11]. Furthermore, intravenous elimination half-life in dogs was as short as 52 min [12]. Thus, various new formulations of scutellarin have been studied to overcome the above disadvantages, but its poor solubility, poor absorption and low bioavailability have not been completely solved until now. Interestingly, some researchers found that scutellarin was mainly absorbed in the form of its hydrolyzed product scutellarein by intestinal [13], and scutellarein was much more easily absorbed with the triple bioavailability, after oral administration of scutellarin and scutellarein in equal amounts [14]. Furthermore, in the clinical trials [15], a large amount of scutellarein was found in urine and plasma after oral administration of breviscapine in subjects, indicating that breviscapine was firstly hydrolyzed into aglycone when reaching colon and was then absorbed as scutellarein as the real bioactive components in the body. Pharmacodynamics confirmed that scutellarein had better protective effect than scutellarin in rat cerebral ischemia [16].

There is little scutellarein in *E. breviscapus* compared with large amount of scutellarin [16]. Frakas [17] and Cui [18] had already completed the total synthesis of scutellarein, but the route was long and the yield was low. As a result, scutellarein cannot be purchased in the market like scutellarin, and it is also very difficult to obtain it through other methods. Thus, we tried the synthesis of scutellarein by hydrolyzing scutellarin in water according to a patent [19]. Unfortunately, it was found that the reactants did not react. As a result, this study was intended to establish an efficient route to the synthesis of scutellarein by hydrolyzing scutellarin, and to preliminarily investigate its antioxidant activity in comparison with scutellarin, which will guide the search for more potent protective agents for ischemic cerebrovascular disease.

## 2. Materials and Methods

### 2.1. Materials

Scutellarin was purchased from Mianning Jiexiang Co. Ltd. (Chengdu, China). 1,1-Diphenyl-2- picrylhydrazyl (DPPH), 2,2′-azinobis[3-ethylbenzothiazoline-6-sulfonicacid]-diammonium salt (ABTS) were purchased from Sigma Chemical Co. (Shanghai, China). Commercial kits used for determination of hydroxyl radical (^•^OH) scavenging activity was purchased from Jiancheng Institute of Biotechnology (Nanjing, China). Dulbecoo’s Modified Eagles Medium (DMEM) was the product of Gibco. Heat-inactivated fetal calf serum was purchased from Sijiqing Institute of Biotechnology (Hangzhou, China). 3-(4,5-Dimethythiazol-2-yl)-2,5-diphenyltetrazolium bromide (MTT) was purchased from Fluka. PC12 cell line was obtained from Institute of Cell Biology, Chinese Academy of Sciences (Shanghai, China).

### 2.2. General Procedure for the Synthesis of Scutellarein

All the reagents were commercially available and used directly. Air- and moisture-sensitive liquids and solutions were transferred via syringe or stainless steel cannula. Organic solutions were concentrated by rotary evaporation below 45 °C at approximately 20 mm Hg. All non-aqueous reactions were carried out under anhydrous conditions using flame-dried glassware within an argon atmosphere in dry and freshly distilled solvents, unless otherwise noted. Reactions were monitored by thin-layer chromatography (TLC) carried out on 0.15~0.20 mm Yantai silica gel plates (RSGF 254) using UV light as the visualizing agent. Chromatography was performed on Qingdao silica gel (160~200 mesh) using petroleum ether (60~90) and ethyl acetate as the eluting solvent.

**Figure 1:**
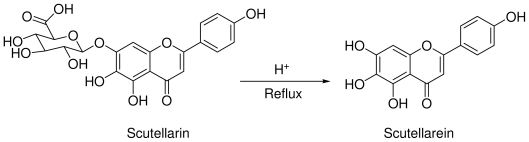
Synthesis of scutellarein.

Scutellarin (0.5 g) was added to 10 mL solution of H_2_SO_4_ in 0~90% ethanol (0.5~3 mol/L). Then the reaction mixture was refluxed at 90~120 °C in N_2_ atmosphere for 6~48 h. After cooling to 25 °C, the reaction mixture was added to ice water; the solid was filtered and then recrystallized with 90% ethanol to give the target compound scutellarein. The synthesis route of scutellarein was outlined in Figure 1. Detailed synthesis conditions were designed in Table 1.

### 2.3. DPPH Radical-Scavenging Activity Assay

The DPPH radical-scavenging activity assay was measured according to the method [20] with a few modifications. 100 μL of the sample (4~250 μmol/L, dissolved in ethanol) was added to 100 μL of ethanol solution containing DPPH radicals (80 μmol/L) in 96 well plates. The mixture was shaken and left for 30 min at room temperature in the dark, and the absorption was measured at 517 nm. Ascorbic acid was used as the positive control. A lower absorbance represents a higher DPPH scavenging activity. The percentage scavenging effect was calculated as scavenging rate (%) = [1 − (*A*_1_ − *A*_2_)/*A*_0_] × 100%, where *A*_0_ was the absorbance of the control (without sample), *A*_1_ was the absorbance in the presence of the sample and *A*_2_ was the absorbance without DPPH.

### 2.4. ABTS^+•^ Radical-Scavenging Activity Assay

The ABTS^+^*^•^* radical-scavenging activity assay was measured according to a literature procedure [21]. ABTS (7 mmol/L) and K_2_S_2_O_8_ (2 mmol/L) were dissolved and mixed in deionized water, reacting for 12~16 h at room temperature. The mixture (ABTS^+^*^•^* stock solution) was then diluted by phosphate buffer solution (PBS) to give an absorbance near 0.7 at 734 nm, defined as the reference absorbance (*A*_0_). *A*_0_ decreased to a stable value (*A*_1_) when 100 μL of ABTS^+^*^•^* was mixed with 100 μL the sample (1~62.5 μmol/L) for 6 min. The percentage scavenging effect was calculated as scavenging rate (%) = [1 − (*A*_1_ − *A*_2_)/*A*_0_] × 100%, where *A*_0_ was the absorbance of the control (without sample), *A*_1_ was the absorbance in the presence of the sample and *A*_2_ was the absorbance without ABTS^+•^.

### 2.5. ^•^OH Radical-Scavenging Activity Assay

The Hydroxyl radical-scavenging activity was measured according to the instruction of the assay kit (Nanjing Jiancheng Co., China). The percentage scavenging effect was calculated as scavenging rate (%) = [1 − (*A*_1_ − *A*_2_)/*A*_0_] × 100%, where *A*_0_ was the absorbance of the control (without sample), *A*_1_ was the absorbance in the presence of the sample and *A*_2_ was the absorbance without ^•^OH.

### 2.6. MTT Assay for PC12 Cell Survival

Protection against oxidative stress in PC12 cells was determined using the method [22] with minor modifications. PC12 cells were cultured in DMEM supplemented with 10% (v/v) heat-inactivated fetal calf serum at 37 °C in a humidified atmosphere of 5% CO_2_. PC12 cells were grown on 96-well plates at a density of 5 × 10^4^ cells/mL (100 μL/well) for 24 h. The protective effect against oxidative stress were tested at different concentrations in three types of experiments: (a) coincubation of PC12 cells with sample and H_2_O_2_; (b) preincubation of cells for 30 min with sample before exposing them to H_2_O_2_; (c) preincubation of cells for 8 h with sample before exposing them to H_2_O_2_. After incubation for 3 h with H_2_O_2_ (400 μmol/L) at 37 °C in CO_2_ incubator, the cells were incubated with the 0.5 mg/mL MTT for 4 h. Then, all culture media were removed and 150 μL of DMSO was added to each well, vigorously shaking for 10 min. Finally, the absorbance was assessed at 517 nm. The inhibiting rate of H_2_O_2_-induced cytotoxicity in PC12 cells was calculated as inhibiting rate (%) = [(*A*_1_ − *A*_2_)/(*A*_0_ − *A*_2_)] × 100%, where *A*_1_ was the absorbance in the presence of sample and H_2_O_2_, *A*_2_ was the absorbance in the presence of H_2_O_2_ (model), *A*_0_ was the absorbance without sample and H_2_O_2_ (normal).

## 3. Results and Discussion

### 3.1. Optimization of Reaction Conditions for the Synthesis of Scutellarein

The optimization of reaction conditions were shown in Table 1. The concentrated sulfuric acid was selected as a catalyst. Firstly, 0.5 g of scutellarin was added to 10 mL of 1 mol/L H_2_SO_4_ in water, and the reaction was taken at 90 °C for 6~24 h in N_2_ atmosphere. However, there was no product; even the concentration of H_2_SO_4_ increased from 1 mol/L (Table 1, run 1) to 3 mol/L (Table 1, run 3), due to the poor water solubility of scutellarein. Then, the solvent of water was changed into ethanol, where scutellarein had good solubility. After several attempts, we found that increasing the concentration of ethanol (Table 1, run 46) accelerated its hydrolysis and the yield increased to 2.1% in 90% ethanol (Table 1, run 6). Subsequently, the concentration of H_2_SO_4_ was optimized, when the concentration of H_2_SO_4_ increased from 1.0 mol/L (Table 1, run 7) to 3.0 mol/L (Table 1, run 9), the yield of scutellarein was improved from 5.3% to 10.0%, and the yield could increase to 12.1% when the reaction time extended to 48 h (Table 1, run 10). Lastly, the exogenous reaction temperature was studied in this reaction. When the reaction temperature was raised from 90 °C (Table 1, Run 10) to 120 °C (Table 1, Run 12), the yield of scutellarein increased from 12.1% to 17.3%. As a result, 3.0 mol/L H_2_SO_4_ in 90% ethanol and in N_2_ atmosphere at 120 °C for 48 h was implemented in the synthesis of scutellarein.

### 3.2. DPPH, ABTS^+•^, ^•^OH Radical-Scavenging Activity

**Figure 2:**
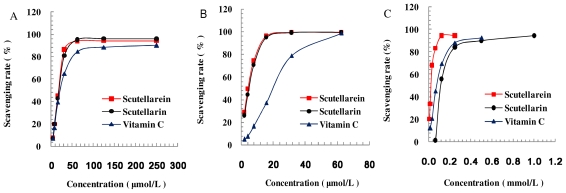
(**A**) The 1,1-Diphenyl-2-picrylhydrazyl (DPPH) radical-scavenging activity assay; (**B**) 2,2′-azinobis[3-ethylbenzothiazoline-6-sulfonicacid]- diammonium salt (ABTS^+•^) radical-scavenging activity assay; (**C**) hydroxyl radical (^•^OH) radical-scavenging activity assay.

DPPH, ABTS^+•^ (both stable radicals) and ^•^OH (generated by Fenton reaction), are widely used to evaluate the antioxidant capacity of complex mixtures and individual compounds [21]. The *in vitro* antioxidant activity of scutellarein in comparison with scutellarin was evaluated by DPPH, ABTS^+•^, ^•^OH radical-scavenging activity assays. The IC_50_ value is defined as the concentration of sample that causes 50% loss of the radical. As illustrated in Figure 2 and Table 2, scutellarein (IC_50_ 16.84 μmol/L for DPPH, 3.00 μmol/L for ABTS^+•^, 0.31 mmol/L for ^•^OH) had stronger radical-scavenging capacities than scutellarin (IC_50_ 17.56 μmol/L for DPPH, 3.53 μmol/L for ABTS^+•^, 3.19 mmol/L for ^•^OH), which indicated that scutellarein is an effective natural antioxidant that might be a promising candidate for ischemic cerebrovascular disease.

### 3.3. Protective Effect on H_2_O_2_-Induced Cytotoxicity in PC12 Cells

PC12 cells can adopt a neuronal phenotype and have been used extensively as a model for catecholamine-secreting neuronal cells [23]. Active mitochondria of living cells can cleave MTT to produce formazan, the amount of which is directly related to the number of living cells. As shown in Table 3, cell viability markedly decreased after PC12 cells were exposed to H_2_O_2_. However, when the cells were incubated with scutellarein or scutellarin, H_2_O_2_-induced cell toxicity was significantly attenuated, and the protective effect of scutellarein was dose-dependent. However, coincubation of cells showed better protective effect than preincubation for some time against H_2_O_2_ cytotoxicity, and the protective effect decreased as preincubation time was prolonged, which may be due to the unstability of scutellarein. Therefore, coincubation was considered to more effectively evaluate cytoprotection of scutellarein; and scutellarein showed a significantly better protective effect than scutellarin. The cellular protective effect of scutellarein might be resulted from its antioxidant action mainly including preventing lipid peroxidation by eliminating radicals [24,25].

## 4. Conclusion

In summary, an efficient route was reported for the synthesis of scutellarein by hydrolyzing scutellarin. Essential to the synthesis was the implementation of H_2_SO_4_ in 90% ethanol in N_2_ atmosphere. The *in vitro* antioxidant assays clearly demonstrated that scutellarein had stronger scavenging capacities toward DPPH, ABTS^+•^, ^•^OH free radicals than scutellarin, and had better protective effect on H_2_O_2_-induced cytotoxicity in PC12 cells. The results suggested that it would be a promising potent agent for the therapy of ischemic cerebrovascular disease. In addition, these findings concluded the need for further study on the pharmacokinetics and its bioactivities other than antioxidant activities.

## Figures and Tables

**Table 1:** Optimization of reaction conditions in the synthesis of scutellarein by hydrolyzing scutellarin.

Run	Reaction conditions	Yield (%)
**1**	1.0 mol/L H_2_SO_4_ in water, 90 °C, 6~24 h	No product
**2**	2.0 mol/L H_2_SO_4_ in water, 90 °C, 6~24 h	No product
**3**	3.0 mol/L H_2_SO_4_ in water, 90 °C, 6~24 h	No product
**4**	0.5 mol/L H_2_SO_4_ in 70% ethanol, 90 °C, 6~24 h	No product
**5**	0.5 mol/L H_2_SO_4_ in 80% ethanol, 90 °C, 6~24 h	No product
**6**	0.5 mol/L H_2_SO_4_ in 90% ethanol, 90 °C, 24 h	2.1
**7**	1.0 mol/L H_2_SO_4_ in 90% ethanol, 90 °C, 24 h	5.3
**8**	2.0 mol/L H_2_SO_4_ in 90% ethanol, 90 °C, 24 h	8.5
**9**	3.0 mol/L H_2_SO_4_ in 90% ethanol, 90 °C, 24 h	10.0
**10**	3.0 mol/L H_2_SO_4_ in 90% ethanol, 90 °C, 48 h	12.1
**11**	3.0 mol/L H_2_SO_4_ in 90% ethanol, 100 °C, 48 h	15.2
**12**	3.0 mol/L H_2_SO_4_ in 90% ethanol, 120 °C, 48 h	17.3

**Table 2:** *In vitro* antioxidant activity of scutellarein in comparison with scutellarin in DPPH assay (IC_50_ in μmol/L), ABTS^+•^ assay (IC_50_ in μmol/L), ^•^OH assay (IC_50_ in mmol/L).

Compounds	IC_50_		

	DPPH (μmol/L)	ABTS^+•^ (μmol/L)	^•^OH (mmol/L)
Scutellarein	16.84	3.00	0.31
Scutellarin	17.56	3.53	3.19
Vitamin C	24.81	12.56	1.12

**Table 3:** Attenuation of H_2_O_2_-induced PC12 cell damage by scutellarein (*x ± s*, *n* = 5).

Drug (μmol/L)	Coincubation		Preincubation for 30 min		Preincubation for 8 h	

	A_517_	Inhibiting Rate (%)	A_517_	Inhibiting Rate (%)	A_517_	Inhibiting Rate (%)
Normal	0.550 ± 0.004	—	0.589 ± 0.003	—	0.624 ± 0.004	—
H_2_O_2_	0.353 ± 0.006 ##	—	0.319 ± 0.0123 ##	—	0.375 ± 0.015 ##	—
Scutellarin/100	0.433 ± 0.009 **	40.78	0.440 ± 0.009 **	44.81	0.482 ± 0.002 **	42.97
Scutellarein/100	0.540 ± 0.038 **	94.92	0.500 ± 0.040 **	67.04	0.458 ± 0.013 **	33.33
Scutellarein/10	0.422 ± 0.007 **	35.32	0.395 ± 0.019 **	28.12	0.414 ± 0.002 **	15.96
Scutellarein/1	0.362 ± 0.002	4.57	0.336 ± 0.007 *	6.10	0.383 ± 0.010	—

** *p* < 0.01,

* *p* < 0.05 *vs*. H_2_O_2_ group;

## *p* < 0.01 *vs*. Normal group.
